# Spatio-temporal malaria transmission patterns in Navrongo demographic surveillance site, northern Ghana

**DOI:** 10.1186/1475-2875-12-63

**Published:** 2013-02-13

**Authors:** Simon Kasasa, Victor Asoala, Laura Gosoniu, Francis Anto, Martin Adjuik, Cletus Tindana, Thomas Smith, Seth Owusu-Agyei, Penelope Vounatsou

**Affiliations:** 1 Swiss Tropical and Public Health Institute, Socinstrasse 57, P.O. Box 4002, Basel, Switzerland; 2 University of Basel, Basel, Switzerland; 3 School of Public Health, Makerere University College of Health Sciences, Kampala, Uganda; 4 Navrongo Health Research Centre, Navrongo, Ghana; 5 School of Public Health, University of Ghana, Legon, Ghana; 6 INDEPTH Network Secretariat, Accra, Ghana; 7 Kintampo Health Research Centre, Ghana Health Services, Ministry of Health, Kintampo, Ghana

**Keywords:** Entomological inoculation rate, Spatio-temporal, Zero-inflated, Malaria, Malaria Transmission Intensity and Mortality Burden Across Africa (MTIMBA) project

## Abstract

**Background:**

The relationship between entomological measures of malaria transmission intensity and mortality remains uncertain. This is partly because transmission is heterogeneous even within small geographical areas. Studying this relationship requires high resolution, spatially structured, longitudinal entomological data. Geostatistical models that have been used to analyse the spatio-temporal heterogeneity have not considered the uncertainty in both sporozoite rate (SR) and mosquito density data. This study analysed data from Kassena-Nankana districts in northern Ghana to obtain small area estimates of malaria transmission rates allowing for this uncertainty.

**Methods:**

Independent Bayesian geostatistical models for sporozoite rate and mosquito density were fitted to produce explicit entomological inoculation rate (EIR) estimates for small areas and short time periods, controlling for environmental factors.

**Results:**

Mosquitoes were trapped from 2,803 unique locations for three years using mainly CDC light traps. *Anopheles gambiae* constituted 52%, the rest were *Anopheles funestus.* Mean biting rates for *An. funestus* and *An. gambiae* were 32 and 33 respectively. Most bites occurred in September, the wettest month. The sporozoite rates were higher in the dry periods of the last two years compared with the wet period. The annual EIR varied from 1,132 to 157 infective bites. Monthly EIR varied between zero and 388 infective bites. Spatial correlation for SR was lower than that of mosquito densities.

**Conclusion:**

This study confirms the presence of spatio-temporal heterogeneity in malaria transmission within a small geographical area. Spatial variance was stronger than temporal especially in the SR. The estimated EIR will be used in mortality analysis for the area.

## Background

Malaria continues to be endemic in most sub-Saharan countries, particularly in Ghana where this study was carried out [1-4]. Malaria in Ghana is transmitted by two main vectors: *Anopheles gambiae* and *Anopheles funestus,* whose peak activities occur at the end of the wet season. Changes in climate, land use and environmental factors profoundly influence the vector, and hence the parasite and transmission patterns. Malaria transmission intensity is measured using clinical (spleen rate), parasitological (parasite infection rate), entomological (entomological inoculation rate [EIR]) or serological markers [5,6]. The most direct measurement of transmission intensity is EIR, the number of infective bites per person per unit time. It is calculated as a product of the proportion of mosquitoes with sporozoite in their salivary glands (sporozoite rate) and numbers of vectors biting an average human in unit time (the human biting rate) [7].

Malaria transmission in sub-Saharan Africa is heterogeneous, varying between climatic seasons, ecological zones and even among areas in close proximity [8-15]. In Ghana, malaria transmission has shown a clear variation over time, season and space [16-18]. The relationship between malaria transmission and mortality is still unclear [19,20]. To clarify the relationship between malaria transmission and mortality, the Malaria Transmission Intensity and Mortality Burden Across Africa (MTIMBA) project was established in 10 INDEPTH network sites between 2001 and 2004 [21,22]. Entomological data were collected every two weeks over a large number of compounds within each site for a period of three years. Each site used a slightly different sampling strategy for mosquitoes depending on available resources and local settlement patterns, aiming to obtain an unbiased estimate of the numbers of biting mosquitoes. These data are spatially correlated because neighbouring compounds share common exposures such as interventions, land use, climate and environmental factors. The longitudinal nature of the data also introduces a temporal correlation.

Rumisha and Amek [23,24] developed geostatistical temporal models to obtain EIR exposure surfaces for the Rufiji and Kisumu MTIMBA-health and demographic surveillance (HDSS) sites, respectively. Subsequent analyses linking mortality to EIR exposure indicated a positive linear relationship between mortality and malaria transmission intensity among the under-fives and a negative association for individuals aged 60 years and above. Although malaria is common in sites, their endemicity, spatio-temporal patterns and mosquito composition are completely different. Malaria transmission in Rufuji is driven by both *An. funestus* and *An. gambiae*, while the later is dominant in Kisumu throughout the year. Kisumu experiences two transmission peaks in a year and Rufiji has only one. This is partly due to ecological differences between the two sites. In relation to breeding sites, *An. funestus* prefer clear, permanent fresh waters while *An. gambiae* larvae are found mostly in temporal and shallow water bodies. Estimating site-specific heterogeneity in malaria transmission will help clarify how variation in transmission influences the malaria-related mortality.

This study reports spatially and temporally explicit estimates of EIR at high resolution, obtained by analysing the MTIMBA data collected from Kassena-Nankana district in northern Ghana where the Navrongo health and demographic surveillance system (NHDSS) is located. The EIR was estimated from Bayesian geostatistical models, fitted separately for sporozoite rate (SR) (assumed to be binomially distributed) and mosquito density data (negative binomially distributed). Model-based predictions at unobserved locations generated spatially explicit and season-specific estimates of EIR for the entire area. These estimates will subsequently be used in addressing the MTIMBA project’s main objective of estimating the relationship of mortality with malaria transmission.

## Methods

### Description of the study area

The NHDSS is located in the administrative district of Kassena-Nankana (between latitude 10° 30^’^ and 11° 00^’^ North and longitude 1° 00^’^ and 1° 30^’^ West), in northern Ghana, bordering Burkina Faso. Its altitude stretches up to 400 m above sea level. The district covers an area of 1,675 sq km and lies within the Guinea savannah belt. Approximately 140,000 people reside in the district and the majority are subsistence farmers. There are two distinct seasons; the wet, between April and October and a dry period that covers remaining months of the year. The region receives approximately 850 mm of precipitation per year with monthly temperatures ranging between 18°C and 45°C. The HDSS routinely collects demographic data using “a compound” as a unit of observation. Malaria is endemic in the area and *Plasmodium falciparum* is transmitted by both *An. gambiae* and *An. funestus*. *Anopheles gambiae* s.s. has previously been reported as a dominant sibling species of the *An. gambiae* complex. The *An. gambiae* M form is predominant in the northern parts of Ghana where NHDSS is located [13,16]. The canals from Tono dam and irrigated lands serve as breeding sites for *An. gambiae* throughout the year, while the rice fields support *An. funestus* breeding especially during the periods when the vegetation is flooded. The small dams that are used in the dry seasons favour mosquito growth in these areas. Malaria transmission in the district occurs throughout the year. Between 2001 and 2002, the recorded mean EIR for the district was as high as 418 infective bites per person per year (ib/p/y) [16]. Further characteristics of the district and the HDSS have been described elsewhere [3,16,25].

### Data types and sources

### Entomological data

Mosquitoes were collected from randomly selected compounds using both light traps and human landing methods following the MTIMBA protocol. Compounds were randomly selected at the beginning of the study using the HDSS database and were allocated to trapping weeks. Sampled compounds were between 100–500 metres apart and were balanced in terms of numbers for the two major zones namely: irrigated and non-irrigated areas. Only one trap was set per compound per night. Light trap catches were performed overnight (from 18:00 GMT to 06:00 GMT). No study team member visited the compound at night until it was time to remove traps the next morning. Such visits were perceived by community members as intrusion. Traps were hung about 1.5 m above the floor next to the bed of an “indexed” person. Heads and thoraces of light-trapped *Anopheles* were tested for *P. falciparum* circumsporozoite protein using enzyme linked immunosorbent assay (ELISA) [26].

The entomological inoculation rate was therefore computed as a product of human biting rate and the proportion of infectious mosquitoes (sporozoite rate). Human biting rate was estimated as a geometric mean of *Anopheles* mosquitoes caught per light trap set [27]. Mosquitoes were trapped in 56% of the 2,803 uniquely georeferenced compounds within the site. Infectious mosquitoes were only found in 28% of these locations.

### Environmental data

Environmental and climatic predictors were obtained from various remote sensing sources. Day and night land surface temperature (LST) at 1 x 1 km and both normalized difference vegetation index (NDVI) plus enhanced vegetation index (EVI) at 250 x 250 m were downloaded from Moderate Resolution Imaging Spectro-radiometer (MODIS). LST and vegetation data were extracted at eight-day and 16-day temporal resolutions respectively. Rainfall estimates (RFE) at 8 x 8 km were obtained at 10-day intervals from the African Data Dissemination Service (ADDS). Altitude at 1 x 1 km was obtained from US Geological Survey (USGS) data centre. Distance to water bodies (based on local rivers and wetlands) was downloaded from HealthMapper version 4.2 databases. The shortest Euclidean distance from water bodies to compounds was calculated using ArcGIS version 9.1 software. The climatic and environmental variables were processed at the locations where entomological data were available. For each location, temperature, rainfall and vegetation data were summarized by month for each year of the project.

### Data analysis

Non-spatial logistic and negative binomial regression models were used to analyse sporozoite and density data respectively. Zero-inflated models were fitted to account for the large number of locations with either no mosquitoes (44%) or no infectious mosquitoes (72%). The Akaike’s information criterion (AIC) in STATA was used to assess the length of the elapsing time (lags) between climatic suitability and malaria transmission. In particular, five summary estimates were computed for each of the environment factors based on mosquito collection month in a year: i) current month of collection, ii) previous month, iii) previous two months, iv) average of the current and previous month, and v) average of the current and previous two months. Three temperature proxies were considered: land surface day, night and average temperature. Seasonality was taken into account by either a binary variable (wet/dry) or trigonometric functions with: (i) one cycle indicating a single transmission season, or (ii) two cycles corresponding to two transmission seasons per year. AIC was used to identify a suitable combination of climatic and environmental predictors for both SR and density by vector species.

Bayesian geostatistical formulations of the above models were fitted to take into account spatio-temporal correlation. In each model, compound-specific random effects were included. They were assumed to be latent observations from a multivariate Gaussian spatial process with a zero mean. The covariance of the process included the spatial variance and an exponential correlation function of distance between any pair of compound locations. First-order autoregressive terms were included to model temporal correlation. Any remaining non-spatial variation (nugget parameter) was considered by an additional set of location random effects, assumed to be mutually independent and normally distributed with zero mean. All the corresponding random and the covariates effects were modelled either on a logit or log scale depending on the model; logistic regression for sporozoite and negative binomial regression for the mosquito density data, respectively. Bayesian kriging was applied to predict SR and mosquito density over a grid of 31,308 pixels with 250 × 250 m spatial resolution. The analysis was carried out for each mosquito species (i.e. *An. funestus* and *An. gambiae*) separately. Mosquito densities were converted to man-biting rates after adjusting for a factor [27]. The indices were multiplied at each location to generate spatially explicit surfaces of EIR for each species. Maps for the total EIR were generated using ArcGIS software. Details of mathematical description for all models used are given in Additional file 1.

### Model validation

Models were fitted on 85% of the locations (training sample) and they were validated on the remaining 15% of locations (test sample). In particular, the model’s predictive ability was assessed by estimating the proportion of test locations correctly predicted within Bayesian credible intervals of probability coverage varying from 1 to 100% [28]. The model with the highest number of correctly predicted locations consistently over the intervals was considered as the one with the best predictive performance.

## Results

### Description of density data

**Figure 1:**
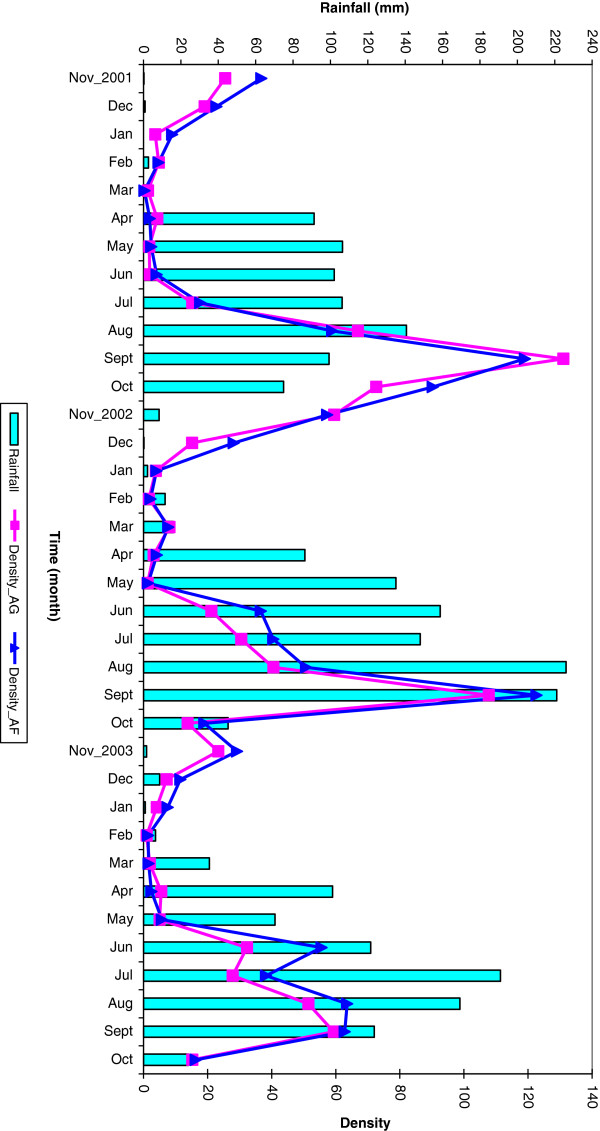
Monthly rainfall and observed mosquito density.

The mean biting rates per person and night for *An. funestus* were 34 in the first year, 32 in the second and 19 in the third. Similarly, *An. gambiae* mean bites were 33 in the first year, followed by 26 and 15 bites in the second and final year respectively. For the entire research period, mean biting rates per month varied with seasonal changes. For both species, most bites were observed during the wet season (July to November). Highest bites occurred in the month of September for all the three years. During the dry period of January to April, fewer monthly bites were recorded. Mosquitoes in the area became more abundant after the first three months of the rainy season (Figure 1).

### Description of sporozoite rate data

**Figure 2:**
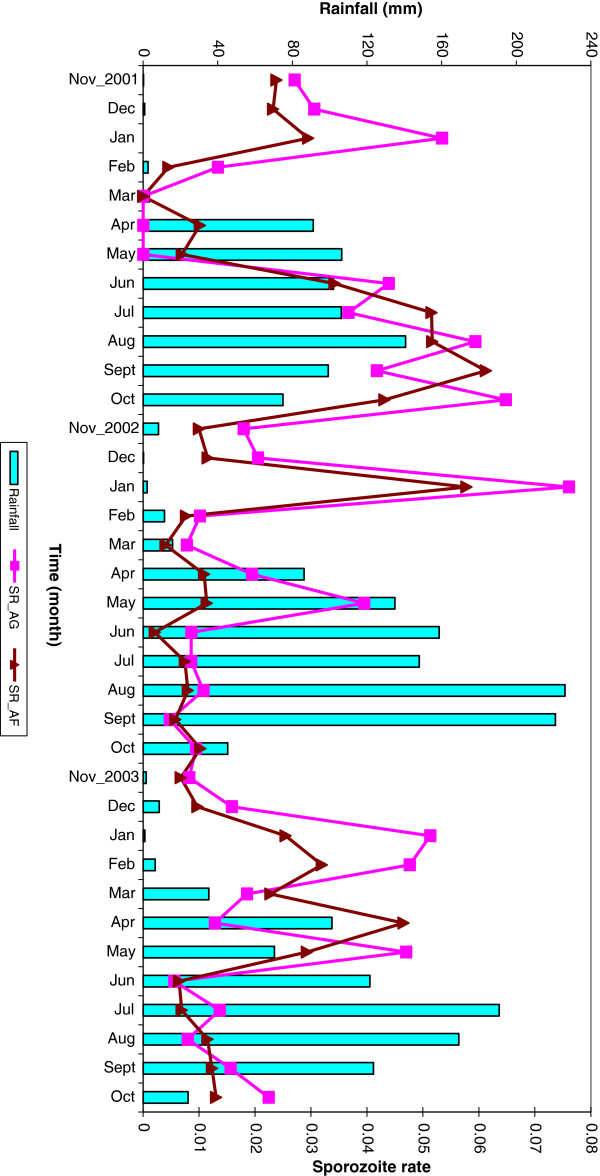
Monthly rainfall and observed sporozoite rate by mosquito species.

A total of 109,647 malaria mosquitoes from 1,565 compounds were tested for sporozoites; 56,887 (52%) were *An. funestus* and the rest were *An. gambiae*. The overall SR was 2.5%. *Plasmodium falciparum* infections were detected in 2.4% of *An. funestus* and 2.7% in *An. gambiae*. The proportion of infectious An*. funestus* was almost equal to that of *An. gambiae* in both the first (4.8% and 4.7%) and third (1.2% and 1.4%) years. The lowest SR of 0.8% was observed in the second year from *An. funestus* mosquito species. The data showed an overall SR of 1.8% and 2.7% in dry and wet season respectively. However, during the second year, the dry period SR was more than double that of wet season (1.6% compared with 0.7%). The proportion of infectious *An. gambiae* (2.1%) was higher than that of *An. funestus* (1.5%) in the dry season*.* The fraction of infected *An. gambiae* mosquitoes was higher in dry season than wet for the second (2.0%) and third (1.5%) year. The monthly SR for both species follows a similar pattern for all the three years (Figure 2).

### Description of entomological inoculation rate data

**Figure 3:**
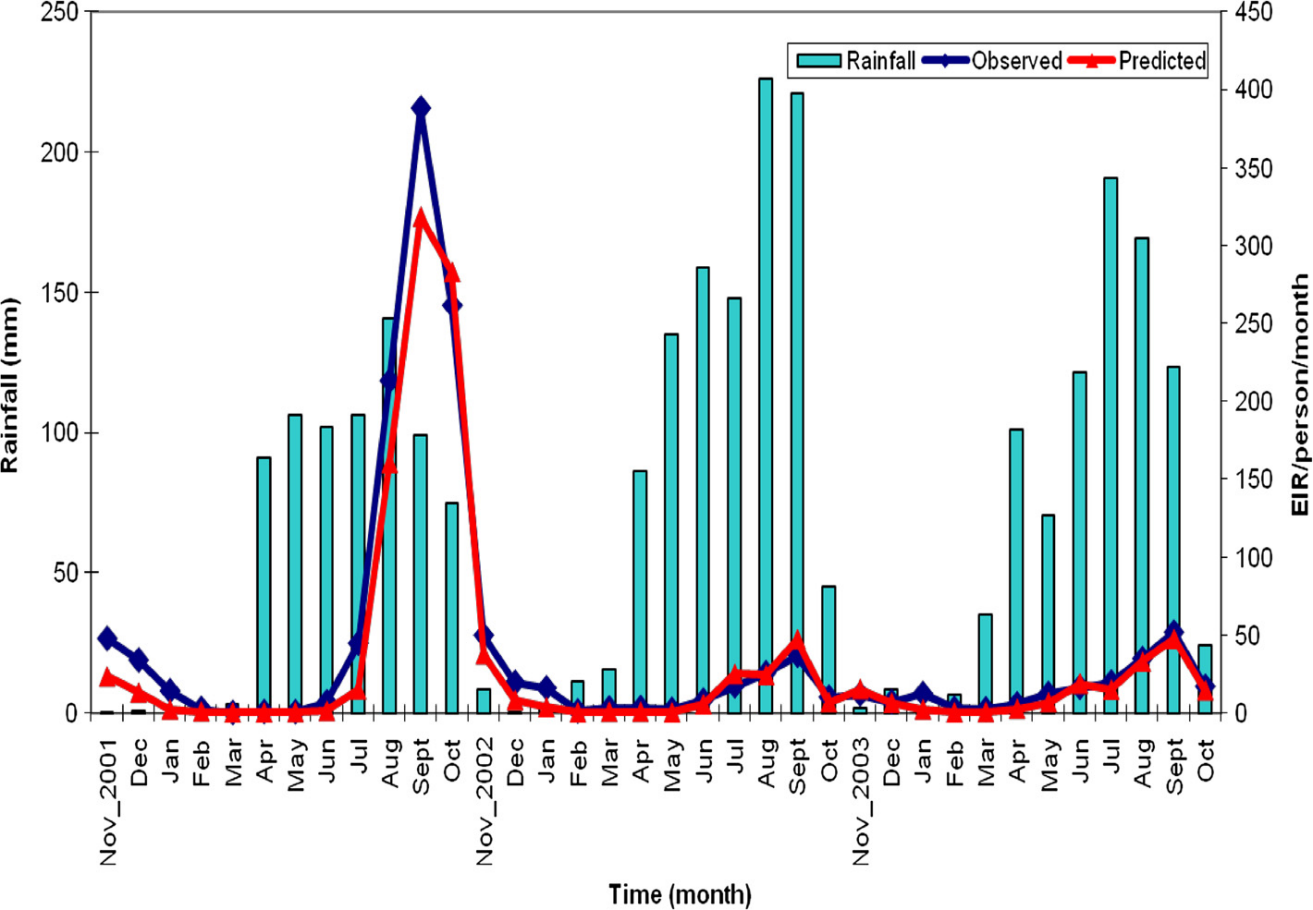
Observed and predicted EIR.

The crude annual EIR estimates, based on entomological data from first to third year were 1132, 193 and 157 ib/p/y respectively (Table 1). The highest EIR was observed in the month of September of each year and varied from 388 in the first year to 37 and 51 infective bites per month in the second and third year respectively. For all the three years, lowest monthly infective bites were observed either in February or March (Figure 3).

**Table 1:** Observed entomological inoculation rate

	**EIR per person per year**		
**Year**	***An. funestus***	***An. gambiae***	**Combined species**
1	575	557	1132
2	90	103	193
3	79	78	157

### Model-based results: mosquito density data

Lag time analysis showed that mosquito density for both species was related to current NDVI, total rainfall, average day and average night temperatures over the two months prior to the survey. Parameter estimates from geostatistical, zero-inflated, negative binomial models are summarized in Table 2. For *An. funestus,* distance to water bodies, NDVI, season, day temperature and second year of data collection were related to density. An increase in vegetation cover was highly associated with an increase in biting rates. Compounds that are close to water bodies were associated with higher number of mosquito bites. Wet seasons and increase in day land surface temperatures were negatively associated with mosquito density. Spatial variation ( $\sigma_{\varphi }^{\left(D\right)2}$ =0.9, (95% CI: 0.5, 1.6)) was almost similar to the temporal one ( $\sigma_{\varepsilon }^{\left(D\right)2}$ =0.82, (95% CI: 0.5, 1.5)).

**Table 2:** Multivariate spatio-temporal analysis for mosquito density by species

**Parameters**	***An. funestus***		***An. gambiae***	
	**Co-efficients**		**Co-efficients**	
	**Median**	**95% CI**	**Median**	**95% CI**
Intercept	2.73	(2.16, 3.31)	1.86	(1.05, 3.67)
Altitude	−0.01	(−0.02, 0.00)	−0.01	(−0.02, 0.00)
Distance to water bodies	−0.12	(−0.22, -0.02)	−0.18	(−0.27, -0.07)
NDVI	2.27	(1.44, 2.83)	1.51	(1.17, 2.39)
Rainfall	0.002	(−0.003, 0.01)	0.0002	(−0.01, 0.01)
Season(Wet)	−0.23	(−0.61, -0.003)	−0.26	(−1.13, 0.33)
Day temperature	−0.04	(−0.09, -0.004)	−0.08	(−0.13, -0.04)
Night temperature	0.08	(−0.02, 0.17)	0.13	(0.04, 0.22)
Year of the survey				
2	−0.98	(−1.33, -0.67)	−0.13	(−1.32, 0.8)
3	−0.74	(−2.51, 1.01)	−0.02	(−1.81, 1.48)
Variances				
Spatial $\left(\sigma_{\varphi }^{\left(D\right)}{}^{2}\right)$	0.94	(0.57, 1.56)	0.87	(0.52, 1.46)
Temporal $\left(\sigma_{\varepsilon }^{\left(D\right)}{}^{2}\right)$	0.82	(0.46, 1.45)	0.88	(0.53, 1.52)
Nugget $\left(\sigma_{e}^{\left(D\right)}{}^{2}\right)$	1.02	(0.75, 1.30)	0.87	(0.61, 1.19)
Range (in km)	38.8	(22.4, 51.0)	38.8	(22.4, 51.0)
Dispersion parameter (r)	0.98	(0.74, 1.17)	0.59	(0.51, 0.70)

For *An. gambiae*, distance to water bodies, NDVI, day temperature and night temperature were associated with mosquito density. Higher day temperatures and longer distances from breeding sources were associated with decline in mosquito density. An increase in vegetation led to an increase in mosquito abundance. Spatial, temporal and non-spatial variances were almost equal. Over-dispersion was present only for *An. gambiae* (r = 0.6, (95% CI: 0.5, 0.7)). The minimum distance at which the spatial correlation was below 5% was 39 km (95% CI: 22.4 km, 51 km) for both species.

### Model-based results: sporozoite rate data

Lag analysis shows that *An. funestus* SR was related to total rainfall of the survey month, average NDVI, average night temperature for the two months preceding the survey, and average day temperature of current and previous month. Similarly, *An. gambiae* SR was driven by the average NDVI of the survey month, total rainfall, and average (of day and night) LST of the current and previous month. Results of SR rate models with spatial and temporal random effects were presented because they provided the best performance with a predictive ability of 40% of the test locations within a 95% Bayesian credible interval. Parameter estimates of the geostatistical logistic regression models are shown in Table 3.

**Table 3:** Multivariate spatio-temporal analysis for sporozoite rate

**Parameters**	***An. funestus***		***An. gambiae***	
	**Co-efficients**		**Co-efficients**	
	**Median**	**95% CI**	**Median**	**95% CI**
Intercept	−0.83	(−2.74, 0.65)	−1.75	(−4.73, 0.04)
Altitude	0.01	(0.002, 0.02)	0.01	( 0.00, 0.02)
Distance to water bodies	−0.22	(−0.33, -0.11)	−0.06	(−0.15, 0.06)
NDVI	−0.6	(−1.30, 0.03)	−0.89	(−1.97, 0.14)
Rainfall	−0.001	(−0.01, 0.004)	−0.01	(−0.01, 0.00)
Season (Wet)	−0.1	(−0.25, 0.06)	0.36	(−0.18, 1.06)
Day temperature	−0.01	(−0.05, 0.04)	-	-
Night temperature	−0.12	(−0.21, -0.03)	-	-
Average temperature	-	-	−0.07	(−0.14, 0.04)
Year of the survey				
2	−0.78	(−1.73, 0.21)	−0.97	(−2.26, 0.17)
3	−0.62	(−1.8, 0.44)	−0.48	(−1.49, 0.28)
Variances				
Spatial ( $\sigma_{\phi }^{{\left(S\right)}^{2}}$)	0.6	(0.40, 0.96)	0.77	(0.56, 1.09)
Temporal $\left(\sigma_{\varepsilon }^{\left(S\right)}{}^{2}\right)$	0.3	(0.15, 0.63)	0.38	(0.18, 0.88)
Range (in km)	4.1	(2.0, 9.2)	2.0	(1.0, 4.1)
Mixing proportion (*π*)	0.54	(0.53, 0.56)	0.54	(0.53, 0.55)

Altitude, distance to the nearest water bodies and night temperature were associated with *An. funestus* SR. Higher night temperatures were associated with low SR in that area. Similarly, places closer to water bodies observed a higher proportion of infectious mosquitoes than others. A positive association between *An. funestus* SR and altitude was estimated. Spatial variability ( $\sigma_{\phi }^{{\left(S\right)}^{2}}$ =0.6, (95% CI: 0.4, 1.0)) was higher than temporal one ( $\sigma_{\varepsilon }^{\left(S\right)}{}^{2}$ =0.3, (95% CI: 0.2, 0.6)). The minimum distance at which the spatial correlation is below 5% was 4.1 km (95% CI: 2.0 km, 9.2 km). On the other hand, altitude was the only factor associated with SR for *An. gambiae*. Spatial variation from *An. gambiae* sporozoite model ( $\sigma_{\varphi }^{\left(S\right)}{}^{2}$ =0.8, (95% CI: 0.6, 1.1)) was twice as high as the temporal one ( $\sigma_{\varepsilon }^{\left(S\right)}{}^{2}$ =0.4, (95% CI: 0.2, 0.9)). The minimum distance at which the spatial correlation is below 5% was 2.0 km (95% CI: 1 km, 4 km). This shows a slower decay of the correlation with distance for the *An. funestus* SR compared with *An. gambiae.*

### Model-based results: entomological inoculation rate estimates

**Figure 4:**
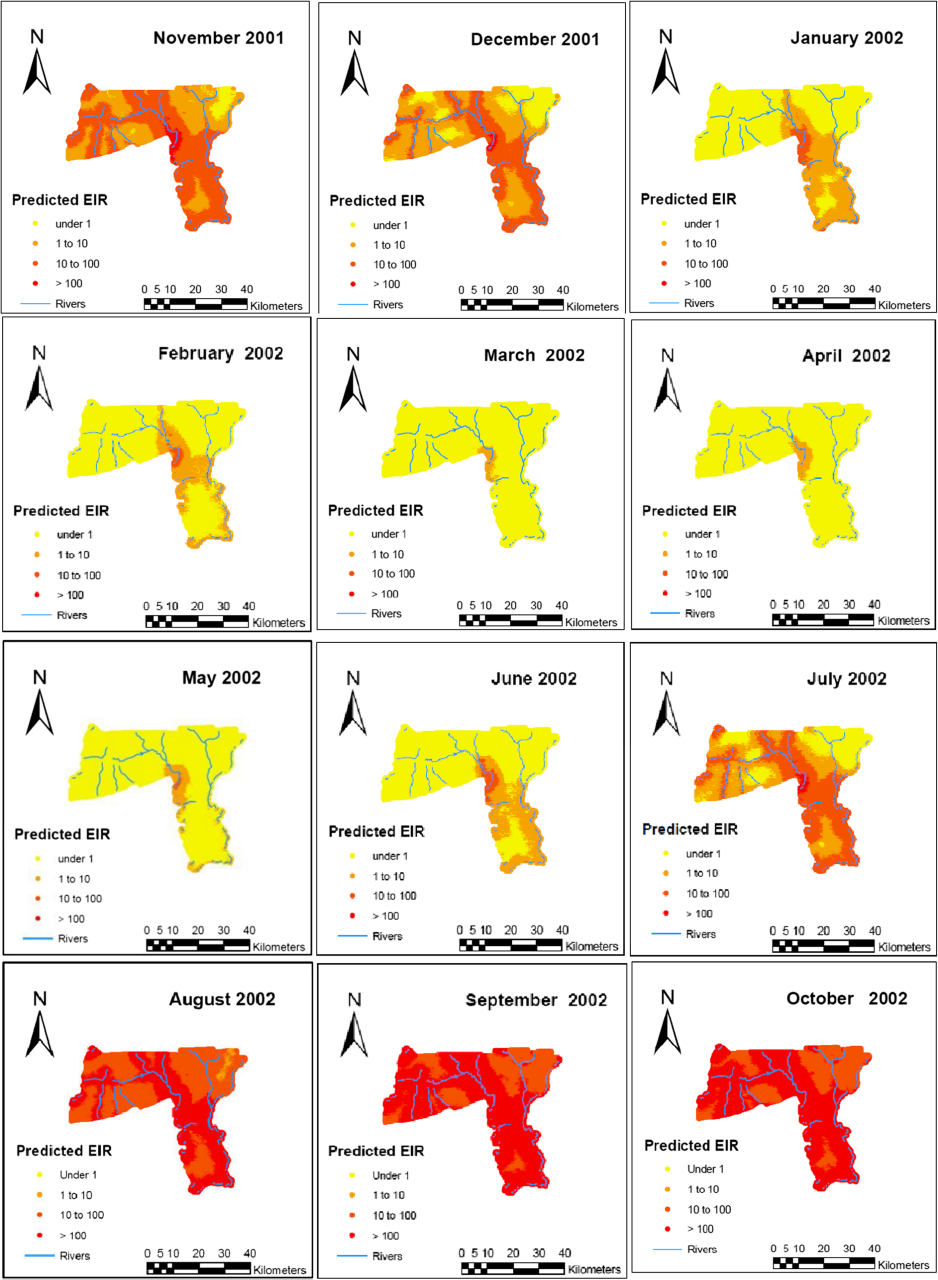
Predicted EIR by month for the first year.

Figure 3 shows the temporal patterns in the EIR values that were captured by the spatio-temporal models. Smooth monthly EIR maps (Figure 4) clearly show a seasonal pattern, ranging from almost no infective bites in the dry season to the highest number of infective bites toward the end of wet season. It is evident from the maps that areas close to water bodies experienced high EIR.

## Discussion

This is the first study assessing malaria transmission heterogeneity in the Navrongo HDSS using a comprehensive entomological dataset and rigorous geostatistical and temporal models, which take into account data characteristics. These data indicate the presence of seasonal, spatial and year-to-year variation within a small geographical area (1,675 km^2^) in northern Ghana. The findings confirm previous studies reporting heterogeneity in malaria transmission in small areas. In particular, spatio-temporal variation has been observed in coastal Kenya [29], in Kilombero valley in Tanzania [15], in some selected Ugandan villages [9] and in a low transmission zone in Sudan [30].

Transmission in the Kassena-Nankana district is high (EIR > 100 ib/p/y) especially during the wet season. An entomological survey conducted in the same district between June 2001 and May 2002 recorded EIR of 630 ib/p/y in the irrigated zone within the southern part of the district [16] which is lower than the one observed in the first year of the MTIMBA project. The drop of EIR after the first year may be explained by variations in laboratory testing. The ELISA tests for the first year were carried out in a different laboratory from those in the remaining two years, making it possible that inter-laboratory differences contribute to inter-annual variation. The year effect included in the model is, therefore, aliased with any laboratory differences. Consequently, there will be more confidence in EIR comparisons between locations than those that depend on inter-annual differences.

This study confirmed the presence of *An. funestus* and *An. gambiae* malaria vector species in the region [16-18], with both acting as major vectors. NDVI, distance to water bodies and temperature were associated with mosquito density for both species. Compounds located close to water bodies were more likely to have high mosquito densities. The Kassena-Nankana district in northern Ghana has many irrigation dams that were constructed to increase food production in the area. There are also many small dugout reservoirs in the area which supply water to various communities especially in the dry season [31,32]. These water bodies can be favourable breeding grounds and responsible for mosquito abundance in neighbouring compounds. The data showed that a reduction in day temperature favoured higher number of mosquito bites in the area. The NHDSS where data were collected experiences high temperatures in some months (18°C to 45°C). Temperatures close to 40°C reduce mosquito survival, hence their density [33]. Although rainfall had a positive relationship with mosquito density, the association was not statistically important. However, rainfall is known to have a direct relationship with other factors, such as vegetation, that were found to positively influence mosquito abundance. A positive correlation between precipitation and mosquito density for both *An. funestus* and *An. gambiae* has already been observed in other places.

A seasonal pattern in mosquito density was observed for both species. High mosquito densities were observed in the rainy season for all the three years and low densities during the dry season. However, SR was higher in the dry than the rainy season during the second and third year. In addition, *An. gambiae* SR in the dry period were higher than that of *An. funestus* for the entire survey period. There was no evidence of variations in SR between species in the rainy season. More infected mosquitoes during dry seasons have already been observed in other areas [14]. This implies that most surviving adult mosquitoes in dry seasons are likely to be infectious.

The shortest distance at which the spatial correlation was below 5% was lower for SR than mosquito densities, suggesting that SR depends largely on local conditions rather than environmental factors. On the other hand, mosquito densities had strong spatial correlation and therefore they are more likely to be driven by environmental factors, especially vegetation which was the major predictor in the Navrongo area. Climate and environmental factors influence malaria transmission and its effects. In this district, malaria illnesses and mortality are observed thought the year with peaks in the wet season [34,35]. Blood transfusion, especially in young children, due to anaemia is more common in the rainy season [36].

The EIR maps clearly depict spatial heterogeneity despite the relative small size of the HDSS. The high EIR estimate in the southern part, which is mainly covered by irrigation dams, has been reported previously [16]. Even during the dry season, transmission in the area remained high. In addition, the geographical pattern of EIR was similar across the three years of the project. The spatial and temporal variances of the mosquito density data accounted for about 33% each out of the total variation. However, SR data explained 67% and 33% of the total variation, suggesting that spatial heterogeneity was twice as high as the temporal one. Although space-time heterogeneity could explain total variation of the SR data, there was a remaining 34% unexplained variation for the densities. In principle, focussed malaria control conducted in the knowledge of these patterns of variation might be more effective than generalized intervention programmes, but no intervention programme is likely to be able to adapt to variations on this scale.

Bayesian geostatistical models are the state-of-art methodology to analyse space and time heterogeneity in malaria transmission and have been used to assess malaria risk using prevalence data [37-41]. However, entomological data have large number of zeros, which cannot be estimated by standard geostatistical models. In particular, the Navrongo data had 44% and 72% of locations with zeros for density and SR, respectively. Entomological data were sparse in the other two MTIMBA sites (i e, Rufiji and Kisumu). This problem was addressed by developing geostatistical zero-inflated formulations of binomial models (GZIB) for analysing SR [42]. Zero-inflated analogues of negative binomial models [22,23] were also applied to take into account excess zeros in the density data. These models were able to improve EIR predictions obtained from standard geostatistical analogues.

The EIR estimates of this study will be used further to analyse the relationship between malaria transmission intensity and mortality as part of the ongoing work for the MTIMBA project.

## Competing interest

The authors declare that they have no competing interests.

## Authors’ contributions

SK analysed, interpreted results and drafted the manuscript. VK was the study entomologist. LG contributed to the analysis and drafting of the manuscript. FA coordinated the field activities. MA and CT participated in sampling and data management. TS gave intellectual content and critically revised the draft. SOA led the site team in all the project activities and also critically reviewed the manuscript. PV conceptualized the analysis design, supervised the process and critically revised the manuscript. All authors read and approved the final manuscript.

## Supplementary Material

Additional file 1 Details of mathematical description for all models used. Click here for file

## References

[B1] WHO World Malaria Report 2009 http://www.who.int/malaria/world_malaria_report_2009/en/index.html

[B2] Carneiro I Roca-Feltrer A Griffin JT Smith L Tanner M Schellenberg JA Greenwood B Schellenberg D Age-patterns of malaria vary with severity, transmission intensity and seasonality in sub-Saharan Africa: a systematic review and pooled analysis PLoS One 2010 5 e8988 10.1371/journal.pone.0008988 20126547 PMC2813874

[B3] Oduro A Koram K Rogers W Atuguba F Ansah A Anyorigiya T Ansah A Anto F Mensah N Hodgson A Nkurumah F Severe falciparum malaria in young children of the Kassena-Nankana District of Northern Ghana Malar J 2007 6 96 10.1186/1475-2875-6-96 17662142 PMC1950879

[B4] Clerk CA Bruce J Greenwood B Chandramohan D The epidemiology of malaria among pregnant women attending antenatal clinics in an area with intense and highly seasonal malaria transmission in northern Ghana Trop Med Int Health 2009 14 688 695 10.1111/j.1365-3156.2009.02280.x 19392740

[B5] The malERA Consultative Group on Monitoring Evaluation and Surveillance: **A research agenda for malaria eradication: monitoring, evaluation, and surveillance** PLoS Med 2011 8 e1000400 21311581 10.1371/journal.pmed.1000400 PMC3026689

[B6] Drakeley CJ Corran PH Coleman PG Tongren JE McDonald SLR Carneiro I Malima R Lusingu J Manjurano A Nkya WMM Lemnge MM Cox J Reyburn H Riley EM Estimating medium- and long-term trends in malaria transmission by using serological markers of malaria exposure Proc Natl Acad Sci USA 2005 102 5108 5113 10.1073/pnas.0408725102 15792998 PMC555970

[B7] Beier JC Killeen GF Githure JI Entomologic inoculation rates and *Plasmodium falciparum* malaria prevalence in Africa Am J Trop Med Hyg 1999 61 109 113 10432066 10.4269/ajtmh.1999.61.109

[B8] Shililu J Ghebremeskel T Mengistu S Fekadu H Zerom M Mbogo C Githure J Novak R Brantly E Beier JC High seasonal variation in entomologic inoculation rates in Eritrea, a semi-arid region of unstable malaria in Africa Am J Trop Med Hyg 2003 69 607 613 14740876

[B9] Okello PE Van Bortel W Byaruhanga AM Correwyn A Roelants P Talisuna A D’Alessandro U Coosemans M Variation in malaria transmission intensity in seven sites throughout Uganda Am J Trop Med Hyg 2006 75 219 225 16896122

[B10] Carter R Mendis KN Roberts D Spatial targeting of interventions against malaria Bull World Health Organ 2000 78 1401 1411 11196487 PMC2560653

[B11] Mabaso MLH Craig M Ross A Smith T Environmental predictors of the seasonality of malaria transmission in Africa: the challenge Am J Trop Med Hyg 2007 76 33 38 17255225

[B12] Kelly-Hope LA McKenzie FE The multiplicity of malaria transmission: a review of entomological inoculation rate measurements and methods across sub-Saharan Africa Malar J 2009 8 19 10.1186/1475-2875-8-19 19166589 PMC2656515

[B13] de Souza D Kelly-Hope L Lawson B Wilson M Boakye D Environmental factors associated with the distribution of Anopheles gambiae s.s in Ghana; an important vector of lymphatic filariasis and malaria PLoS One 2012 5 e9927 20360950 10.1371/journal.pone.0009927 PMC2847902

[B14] Charlwood JD Kihonda J Sama S Billingsley PF Hadji H Verhave JP Lyimo E Luttikhuizen RC Smith T The rise and fall of *Anopheles arabiensis* (Diptera: Culicidae) in a Tanzanian village Bull Entomol Res 1995 85 37 44 10.1017/S0007485300051993

[B15] Drakeley C Schellenberg D Kihonda J Sousa CA Arez AP Lopes D Lines J Mshinda H Lengeler C Schellenberg JA Tanner M Alonso P An estimation of the entomological inoculation rate for Ifakara: a semi-urban area in a region of intense malaria transmission in Tanzania Trop Med Int Health 2003 8 767 774 10.1046/j.1365-3156.2003.01100.x 12950662

[B16] Appawu M Owusu-Agyei S Dadzie S Asoala V Anto F Koram K Rogers W Nkrumah F Hoffman SL Fryauff DJ Malaria transmission dynamics at a site in northern Ghana proposed for testing malaria vaccines Trop Med Int Health 2004 9 164 170 10.1046/j.1365-3156.2003.01162.x 14728621

[B17] Abonuusum A Owusu-Daako K Tannich E May J Garms R Kruppa T Malaria transmission in two rural communities in the forest zone of Ghana Parasitol Res 2010 6 1465 1471 21153839 10.1007/s00436-010-2195-1

[B18] Dery DB Brown C Asante KP Adams M Dosoo D Amenga-Etego S Wilson M Chandramohan D Greenwood B Owusu-Agyei S Patterns and seasonality of malaria transmission in the forest-savannah transitional zones of Ghana Malar J 2010 9 314 10.1186/1475-2875-9-314 21054895 PMC2989982

[B19] Smith TA Leuenberger R Lengeler C Child mortality and malaria transmission intensity in Africa Trends Parasitol 2001 17 145 149 10.1016/S1471-4922(00)01814-6 11286800

[B20] Gemperli A Vounatsou P Kleinschmidt I Bagayoko M Lengeler C Smith T Spatial patterns of infant mortality in Mali: the effect of malaria endemicity Am J Epidemiol 2004 159 64 72 10.1093/aje/kwh001 14693661

[B21] Abdullah S Adazu K Masanja H Diallo D Hodgson A Ilboudo-Sanogo E Nhacolo E Owusu-Agyei S Thompson T Smith T Binka FN Patterns of age-specific mortality in children in endemic areas of sub-Saharan Africa Am J Trop Med Hyg 2007 77 99 105 18165480

[B22] Amek N Bayoh N Hamel M Lindblade KA Gimnig J Odhiambo F Laserson KF Slutsker L Smith T Vounatsou P **Spatial and temporal dynamics of malaria transmission in rural Western Kenya** Parasit Vectors 2012 5 86 10.1186/1756-3305-5-86 22541138 PMC3464956

[B23] Rumisha SF Modelling the seasonal and spatial variation of malaria transmission in relation to mortality in Africa 2011 University of Basel PhD thesis

[B24] Amek NO Bayesian spatio-temporal modelling of the relationship between mortality and malaria transmission in rural western Kenya 2012 PhD thesis. University of Basel

[B25] INDEPTH Network Population and Health in Developing Countries 2002 Canada: Published by International Development Research Centre 247 256

[B26] Wirtz RA Zavala F Charoenvit Y Campbell GH Burkot TR Schneider I Esser KM Beaudoin RL Andre RG Comparative testing of monoclonal antibodies against *Plasmodium falciparum* sporozoites for ELISA development Bull World Health Organ 1987 65 39 45 3555879 PMC2490858

[B27] Lines JD Curtis CF Wilkes TJ Njunwa KJ Monitoring human-biting mosquitoes (Diptera: Culicidae) in Tanzania with light-traps hung beside mosquito nets Bull Entomol Res 1991 81 77 84 10.1017/S0007485300053268

[B28] Gosoniu L Vounatsou P Sogoba N Smith T Bayesian modelling of geostatistical malaria risk data Geospat Health 2006 1 127 139 18686238 10.4081/gh.2006.287

[B29] Mbogo CM Mwangangi JM Nzovu J Gu W Yan G Gunter TJ Swalm C Keating J Regens JL Shililu JI Githure JI Beier JC Spatial and temporal heterogeneity of Anopheles mosquitoes and *Plasmodium falciparum* transmission along the Kenyan coast Am J Trop Med Hyg 2003 68 734 742 12887036

[B30] Hamad AA Nugud AEHD Arnot DE Giha HA Abdel-Muhsin A-MA Satti GMH Theander TG Creasey AM Babiker HA Elnaiem D-E A marked seasonality of malaria transmsission in two rural sites in eastern Sudan Acta Trop 2002 83 71 82 10.1016/S0001-706X(02)00059-1 12062795

[B31] Binka FN Indome F Smith T Impact of spatial distribution of permethrin-impregnated bed nets on child mortality in rural northern Ghana Am J Trop Med Hyg 1998 59 80 85 9684633 10.4269/ajtmh.1998.59.80

[B32] Owusu-Agyei S Smith T Beck H Amenga-Etego L Felger I Molecular epidemiology of *Plasmodium falciparum* infections among asymptomatic inhabitants of a holoendemic malarious area in northern Ghana Trop Med Int Health 2002 7 421 428 10.1046/j.1365-3156.2002.00881.x 12000651

[B33] Craig MH Snow RW le Sueur D A climate-based distribution model of malaria transmission in sub-Saharan Africa Parasitol Today 1999 15 105 111 10.1016/S0169-4758(99)01396-4 10322323

[B34] Binka FN Morris SS Ross DA Arthur P Aryeetey ME Patterns of malaria morbidity and mortality in children in northern Ghana Trans R Soc Trop Med Hyg 1994 88 381 385 10.1016/0035-9203(94)90391-3 7570811

[B35] Koram KA Owusu-Agyei S Utz G Binka FN Baird JK Nkrumah FK Severe anemia in young children after high and low malaria transmission seasons in the Kassena-Nankana district of northern Ghana Am J Trop Med Hyg 2000 62 670 674 11304052 10.4269/ajtmh.2000.62.670

[B36] Owusu-Agyei S Fryauff DJ Chandramohan D Koram KA Binka FN Nkrumah FK Utz G Hoffman SL Characteristics of severe anemia and its association with malaria in young children of the Kassena-Nankana District of northern Ghana Am J Trop Med Hyg 2002 67 371 377 12452491 10.4269/ajtmh.2002.67.371

[B37] Gemperli A Sogoba N Fondjo E Mabaso M Bagayoko M Briët OJT Anderegg D Liebe J Smith T Vounatsou P Mapping malaria transmission in West and Central Africa Trop Med Int Health 2006 11 1032 1046 10.1111/j.1365-3156.2006.01640.x 16827704

[B38] Hay SI Guerra CA Gething PW Patil AP Tatem AJ Noor AM Kabaria CW Manh B Elyazar IRF Brooker S Smith DL Moyeed RA Snow R A world malaria map: *Plasmodium falciparum* endemicity in 2007 PLoS Med 2009 6 e1000048 19323591 10.1371/journal.pmed.1000048 PMC2659708

[B39] Ashton RA Kefyalew T Tesfaye G Pullan RL Yadeta D Reithinger R Kolaczinski JH Brooker S School-based surveys of malaria in Oromia Regional State: Ethiopia: a rapid survey method for malaria in low transmission settings Malar J 2011 10 25 10.1186/1475-2875-10-25 21288368 PMC3039636

[B40] Riedel N Vounatsou P Miller JM Gosoniu L Chizema-Kawesha E Mukonka V Steketee RW Geographical patterns and predictors of malaria risk in Zambia: Bayesian geostatistical modelling of the 2006 Zambia national malaria indicator survey (ZMIS) Malar J 2010 9 37 10.1186/1475-2875-9-37 20122148 PMC2845589

[B41] Giardina F Gosoniu L Konate L Diouf MB Perry R Gaye O Faye O Vounatsou P Estimating the burden of malaria in Senegal: Bayesian Zero-inflated binomial geostatistical modeling of the MIS 2008 data PLoS One 2012 7 e.0032625 10.1371/journal.pone.0032625 PMC3293829 22403684

[B42] Amek N Bayoh N Hamel M Lindblade KA Gimnig J Laserson KF Slutsker L Smith T Vounatsou P Spatio-temporal modeling of sparse geostatistical malaria sporozoite rate data using a zero inflated binomial model Spat Spatiotemporal Epidemiol 2011 2 283 290 10.1016/j.sste.2011.08.001 22748226

