# Supplementary material for: Spatio-temporal malaria transmission patterns in Navrongo demographic surveillance site, northern Ghana
Source: Malar J. 2013 Feb 13;12:63. doi: 10.1186/1475-2875-12-63 (PMC3618087; doi:10.1186/1475-2875-12-63)
Supplement: Additional file 1 — Details of mathematical description for all models used. [file 1475-2875-12-63-S1.doc]

**Additional file 1**

**Spatio-temporal modelling of sporozoite rate**

Sporozoite rate are binomial data modelled via logistic regression. Assume that for a specific species, the number of positive mosquitoes $Y_{it}^{(S)}$ out of all tested $(N_{it})$ follows a binomial distribution; that is $Y_{it}^{(S)}\sim Bin\left(N_{it,}p_{it}\right)$ with parameter$p_{it}$, the SR at location $i$ and time$t.$ To account for spatial and temporal variation in the data, compound and monthly random effects $\varphi_{i}^{(S)}$ and $\varepsilon_{t}^{(S)}$respectively were introduced. These were modelled together with covariates $X_{it}$ on a logit scale as $\log it(p_{it})=X_{it}^{T}\beta^{(S)}+\varphi_{i}^{(S)}+\varepsilon_{t}^{(S)}+e_{i}^{S}$, where $\beta^{(S)}=\left(\beta_{1},\beta_{2},.......,\beta_{k}\right)^{T}$ is a vector of regression co-efficients. The spatial random effects $(\varphi_{i}^{(1)})$are assumed to be latent observations from a Gaussian spatial process with zero mean and variance-covariance matrix$\Sigma^{(S)}$, where$\varphi_{i}^{(S)}=\left(\varphi_{1},\varphi_{2},.......,\varphi_{n}\right)^{T}\sim N(0,\Sigma^{(S)})$. The covariance between any particular pair of locations was considered to be a function of distance between the locations, that is where $d_{ij}^{(S)}$is the Euclidean distance between locations i and j, is the spatial variance and $\rho^{(S)}$is the correlation decay parameter with a range defined as$3/\rho^{(S)}$. Temporal random effects were modelled by a first order autoregressive process [AR (1)] with variance, which allows correlation between consecutive time periods [43]. Non-spatial random effects $e_{i}^{(S)}$with zero mean and variance were added to the model in order to account for unexplained variation in the data. Seasonality variable dry/wet was included in the model also as a covariate.

**Spatio-temporal modelling of mosquito density**

Let the number of mosquitoes $Y_{it}^{(D)}$ caught at location i and time t follows a negative binomial distribution,$Y_{it}^{(D)}\sim NB\left(\mu_{it},r\right)$, with $\mu_{it}$ the mean and $r$, the over-dispersion parameter [44]. Location ($\varphi_{i}^{(D)}$) and monthly ($\varepsilon_{t}^{(D)}$) random effects were introduced to account for spatial and temporal variation in the data. Non-spatial random effects $e_{i}^{(D)}$with zero mean and variance were added to the model in order to account for unexplained variation in the data. The relationship between mean density of each species$(\mu_{it})$, the covariates$X_{it}$ and the random effects is modelled as;

$\log(\mu_{it})=X_{it}^{T}\beta^{(D)}+\varphi_{i}^{(D)}+\varepsilon_{t}^{(D)}+e_{i}^{(D)}$ where, $X_{it}$is the vector of covariates at location i for time t and $\beta^{(D)}=\left(\beta_{1},\beta_{2},.......,\beta_{k}\right)^{T}$, the vector of regression coefficients. Like seasonality, spatial and temporal random effects are defined and modelled in similar way as described in the spatio-temporal model of SR above.

**Zero-inflated models**

The NHDSS entomological data had many locations with either no mosquitoes (56%) or uninfected mosquitoes (72%). This calls for zero-inflated models that add extra weight to the probability of observing zero [45, 46]. Such models have two components; one arising from either binomial or negative binomial distribution and another for excess zero that cannot be estimated by the model. In such models, $\pi{}_{it}$ is the mixing proportion and the corresponding $\left(1-\pi_{it}\right)$is the probability of observing an outcome arising from either binomial or negative binomial distribution. The model therefore is written as follows;

$$P(Y_{it}=y_{it})=\left\{_{(1-\pi_{it})f(y_{it}), y_{it}>0}^{\pi_{it}+(1-\pi_{it})f(y_{it}), y_{it}=0}\right.$$

where $f(y_{it})$is the binomial or negative binomial density function depending on the outcome data.

Models were fitted assuming that either the mixing proportion is constant throughout space and time, i e, $\log it(\pi_{it})=\beta_{0}^{(m)}$ or that it is a function of environmental covariates (NDVI/EVI, LST, and rainfall); i e, $\log it(\pi_{it})=X_{it}^{T}\beta^{(m)}$ , where $X_{it}^{T}$ is the set of covariates and $\beta^{(m)}=\left(\beta_{0},\beta_{1},\beta_{2},\beta_{3}\right)^{T}$ is the vector of regression co-efficients for each mosquito species. Based on model validation, SR data were fitted with constant value, while density with a mixing proportion derived from a function of covariates.

**Model fit and implementation**

Bayesian inference was applied by combining likelihood function and prior distributions to form the posterior distribution that was used in estimating model parameters. Prior distributions specified for all model parameters were; normal with zero mean and large variance for regression coefficients$((\beta^{(1)},\beta^{(D)},\beta^{(S)}); \beta\sim N(0,10^{2}))$, inverse gamma for the variances$((\sigma_{\varphi}^{2},\sigma_{\varepsilon}^{2},\sigma_{e}^{2}); \sigma^{2}\sim IG(2.01,1.01))$, gamma for the dispersion parameter$(r\sim G(0.01,0.01))$, uniform for decay parameter $(\rho\sim U(a,b))$and beta distribution for the mixing proportion$(\pi\sim Be(1,1))$.

Markov Chain Monte Carol (MCMC) simulation algorithm was used in estimating the model parameters. A a single chain sampler of 250000 iterations with an initial burn-in of 10,000 was used. OpenBUGS version 3.1.1 software was used for parameter estimation. FORTRAN program written by the authors was used for Bayesian Kriging in order to predict both SR and density at locations where data were not collected [47].

**Space time prediction of entomological inoculation rate**

Model-based products for SR and density at high resolution were combined to generate EIR. These models were also used to predict EIR at unobserved locations over a grid of 31,308 pixels at a 250 m^2^ spatial resolution.
